# Medication errors and adverse drug events in peri-operative pediatric anesthetic care over twenty years: a retrospective observational study

**DOI:** 10.1186/s12871-025-03109-8

**Published:** 2025-05-15

**Authors:** Shemila Abbasi, Muhammad Azhar Sharafat, Fauzia Khan

**Affiliations:** Department of Anaesthesiology, Aga Khan University, Stadium Road, P.O Box 3500, Karachi, 74800 Pakistan

**Keywords:** Anesthesia, Adverse drug events, Critical incident review, Quality improvement, Pediatrics, Medication error

## Abstract

**Background:**

Children are at an increased risk of medication errors (MEs) during perioperative care compared to adult patients. This study aimed to critically look at medication errors and determine the frequency of adverse drug events and corrective measures taken for medication errors reported over 20 years in pediatric anesthetic care in the anesthesia department of a tertiary care teaching institution in a lower middle-income country (LMIC).

**Methods:**

Two investigators conducted a retrospective review of all critical incident forms received between January 2001 and December 2020 and identified medication errors related to patients aged 18 years or less. In the second phase of the audit, these medication errors were assessed in detail and adverse drug events were identified using a standardized protocol. We also analyzed the strategies that were employed to prevent such incidents in the future.

**Results:**

One hundred and ninety-six pediatric medication errors were identified. 40% of errors were reported in children between 13 and 72 months of age and 58% at induction. The majority of events took place during administration, preparation, and dispensing i.e., 45%, 41%, and 6% respectively. The adverse drug events occurred in 27 (1.2%) reports and life-threatening events in only one report.

**Conclusion:**

13% of the medication errors progressed to adverse drug events (ADE) and half of those were serious and life-threatening. Reinforcement of standard practice in departmental critical incident meetings, patient safety workshops and lessons to learn e-mails were some low-cost strategies to enhance medication safety during anesthesia.

## Background

Pediatric patients present a higher risk for medication errors (MEs) compared with adults [1]. There are several reasons contributing to this in published literature like lack of proper training among professionals, illegible prescriptions, use of abbreviations in prescriptions, fatigue among professionals, inconsistencies in different formulations of available drugs, language barrier, and lack of good communication skills [2–4].

The pediatric medication process is complex and error-prone because of the multiple steps required in calculating, verifying, preparing, and administering doses [5]. There are different phases of drug handling that involve humans from planning to execution with verbal and nonverbal methods of communication [6]. The most important phase that has a high harm rate in this population is the phase of administration if the incident is not recognized or intervened. Human factors are the highest contributor in most of the reports [7]. Human factors also show many categories and are very important in solving the root cause.

We have a departmental anonymous Critical Incidents Reporting System (CIRS) in place for several years. It is open to all anesthesia trainees, consultants, and anesthesia technicians. A previous report that looked at pediatric critical incidents between 1997 and 2002 reported that one-fifth of incidents were related to medication [8].

Our primary objective in doing this study was to critically look at the reported frequency of ME and the frequency of adverse drug events in pediatric patients reported over the last 20 years at a tertiary care hospital. Our secondary objective was to review the corrective measures taken for these MEs at our institution.

## Materials and methods

This was a retrospective observational study conducted at the Department of Anesthesiology at a tertiary care hospital. The Ethical Review Committee (ERC) of the University waived informed consent for this study (ERC no. 2022-3421-20389) and it was conducted according to the Helsinki Declaration of 1975 (revised 2013).

Critical incident (C.I.) reporting system is in place in our department since 1996. The structured CI forms are available in all the operating rooms of the hospital. At the beginning of postgraduate training, the CI reporting system is part of department orientation. The anonymity is maintained by the fact that the identity of the patient, reporting person, date, time, and location are not reported in the form. Filled forms are dropped in a dedicated locked “CI box” placed in the recovery room, which also ensures anonymity. It can be filled either by the medical or allied health staff, including anesthesia trainees, consultants, and technicians. Forms are periodically collected and reviewed by the CI group, and all data variables are entered in an electronic departmental database on Statistical Package of Social Sciences Version 19.0 (SPSS ver-19). The approved improvement measures are shared in the departmental academic meetings. In our study all CI reported from 1st January 2001 until December 2020 were reviewed by two of the authors to retrieve all medication errors reported in patients aged equal to or less than 18 years. The data was then extracted and reviewed further according to a standardized protocol which also provided definitions and classification of drug errors. The extracted data was then reviewed with contextual details independently by the same two authors. Both filled out a pre-designed data extraction form. Any form that does not meet inclusion criteria like lack of proper contextual details was excluded from the study. In case of disagreement on any derived data between the two reviewers, the third investigator was consulted for resolution. In case of disagreement by third investigator that incident was decided as a dropout from the study.

In addition to demographic information, the phase of drug handling, category of administration, error type, class of medication, level of harm, severity in adverse drug event (ADE), and immediate and late steps taken or planned for improvement were also noted.

The phase of drug handling were classified as errors in medication selection, ordering, dispensing, preparing/administering, documenting, and monitoring. These categories are further classified for the sake of understandability. The categories of administration errors were marked using the classification described in previous publications as omission (a drug not administered or administered late), substitution (incorrect drug administered instead of intended drug), repetition (extra dose of an intended drug given), incorrect dose (incorrect concentration, amount, or rate of infusion of the drug administered), insertion (drug administered that was not attended at that time or any stage), and incorrect route [9, 10].

Further classification of error type into equipment, human, and system errors was noted as marked by the primary reporting anesthesiologist. The level of harm was divided from the contextual details as harm or no harm to the patient. ADEs were also further categorized into minor physiological disturbance (significant errors), major physiological disturbance (serious), and morbidity or mortality (life-threatening) in literature [11].

### Statistical analysis

Data was entered and analyzed in S.P.S.S. ver-19.0. Descriptive analysis was carried out to report categories and types of error. Frequencies and percentages were used to report demographic data, medication errors, phase of drug handling, category of administration, error type, class of medication, level of harm, and severity of adverse drug event (ADE).

## Results

During the study period 22,685 pediatric surgeries performed under general anesthesia. A total of 2249 critical incidents were reported in the system between 2001 and 2020 in pediatric patients (age 18 years or less) during their anesthetic management. Our initial review identified 214 medication errors. Eighteen forms were excluded for not meeting the criteria based on the operational definitions in the protocol and due to a lack of contextual details. One hundred and ninety-six medication errors were analyzed.

Age groups, surgical discipline, and phase of anesthesia are shown in Table 1. Medication errors involved 13 different drug categories administered in the perioperative period. Major drug categories were 44 (22.5%) incidents related to neuro-muscular blockers, 39 (20%) opioids, 30 (15.3%) sedative/hypnotics, 26 (13.3%) antibiotics, 14 (7%) paralytic reversal agent and 9 (5%) local anesthetics.

**Table 1:** Patient age, surgical discipline, and phase of anesthesia in pediatric MEs

Variables	Categories	f (%) (*n* = 196)
Age groups	less than 1 month	4 (2%)
	1–12 months	57 (29.1%)
	13–72 months	78 (39.8%)
	More than 72 months	57 (29.1%)
Surgical discipline	Pediatric Surgery	121 (61.7%)
	Neurosurgery	41 (20.9%)
	ENT	20 (10.2%)
	General /Plastic surgery	5 (2.5%)
	Orthopedic	2 (1%)
	Urology	2 (1%)
	Eye	2 (1%)
	Cardiothoracic &Vascular	2 (1%)
Phase of Anesthesia	Preoperative bay	4 (2%)
	Pre induction	7 (3.6%)
	Induction	113 (57.7%)
	Maintenance	51 (26%)
	Emergence	21 (10.7%)

Regarding the phase of handling 88 (45%) incidents were reported during administration, 80 (41%) during preparation, and 12 (6%) during dispensing. Ordering, documentation, planning, and selection, contributed much less, 5 (2.5%), 5 (2.5%), 5 (2.5%), and 1 (0.5) respectively. The most commonly occurring incidents from major contributing categories, their frequency (%), and action for system improvement are shown in Table 2. Percentages were calculated from the total number of medication errors. Out of 88 administration errors, 31 were repetitions, 29 were incorrect doses, 20 were substitutions, four were incorrect routes and four were reported as omissions. One-third (30%) of the administration errors resulted in harm to the patient.

**Table 2:** The cause, immediate and late corrective measures taken in life-threatening and serious ADEs

Age	Type of ADEs	Incident	Cause	Immediate effect/ action	Corrective measures
**Life-threatening ADEs**					
2 Y	Wrong Drug	Bradycardia up to 60/min and apnoea due to muscle paralysis	Post-call resident relieved another resident and mixed Atracurium with neostigmine instead of Atropine	Atropine given Intubation/ ventilation done	From 2004 post post-call duties in the OR were discouraged. Working hours of residents were ensured to meet standards.
**Serious ADEs**					
4Y	STD	Prolonged apnoea after Propofol	A high dose of propofol given without calculation	Ventilation	Calculation of doses before the procedure is a must in pediatrics*
2Y	STD	Overdose of Mannitol resulted in a continuous drop in BP	The drip attached from the ER was not discontinued	-Mannitol discontinued -Fluid and vasopressors used to support hemodynamic parameter	Check every infusion when a patient is transferred from the ER or ICU.
3 Y	STD	Neostigmine given without atropine resulted in Bradycardia up to 70/min	Syringe swap; Two syringes were prepared one with diluted Neostigmine and another syringe with (N + Atropine). Wrong syringe used	Atropine was given and HR improved.	Medications should be prepared at a dedicated place. Leftover medications must be discarded according to policy.
10 M	STD	Bradycardia, apnoea, and cyanosis occurred just after extubation.	10 micrograms of Fentanyl I/V were given for pain. The patient had already received a caudal with Inj. Paracetamol	Reintubation and ventilation	Titration of medication in the pediatric population and reduced doses in multimodal analgesics is crucial*
3 M	STD	Delayed recovery from muscle relaxant which took more than 2 h	Unpredictable duration of action of action of Rocuronium	The patient was kept intubated, then reversed.	Awareness created for its cautious use, in children with metabolic problems*
4 Y	STD	Forehead laceration as the head is struck into a wheelchair	A high dose of Midazolam was given to a child who was shifted on a wheelchair instead of a stretcher	Dressing done shifted to stretcher	Premedication doses must be calculated cautiously for daycare surgeries, especially in pediatrics.
22 D	STD	0.7 mg Atropine given instead of 0.07 mg resulted in tachycardia	Deviation from drug dilution guidelines	Tachycardia settled in a few minutes	Adherence to pediatric drug dilution guidelines*
2 Y	SbTD	Bradycardia up to 75/min	A lower dose of Atropine was calculated and mixed with Neostigmine	I/V Atropine given	Adherence to pediatric drug dilution guidelines*
11 Y	SbTD	A patient bucked on LMA	Empty vaporizer of Isoflurane	Propofol given	Machine check by anesthetist was stressed*
3 Y	SbTD	Laryngospasm at induction	The airway was manipulated in a light plane of anesthesia due to a lower dose of Propofol.	Propofol repeated with 100% oxygen	Calculation of doses before the procedure is a must in pediatrics*
5 M	SbTD	Laryngospasm and desaturation up to 85%	Paracetamol suppository inserted at an inadequate depth of anesthesia	Intubation was done with propofol and succinylcholine	Inj. Paracetamol was added to the formulary. Depth of anesthesia must be ensured before inserting the suppository
1 Y	Non-adherence	Laryngospasm and desaturation up to 47%.	-History of upper respiratory infection -Laryngoscopy at an inadequate depth of anesthesia	Intubation done without muscle relaxant	Succinylcholine and Atropine must be diluted before administration in pediatric cases*
11 Y	Wrong drug	At induction Neostigmine 5 mls given in place of muscle relaxant (Atracurium)	Neostigmine prepared and kept with other medicines. Syringe swap due to same size syringe & labels prepared with paper tape	HR dropped from 115/min to 75/min. Atropine administered	Reversal preparation at the end of surgery Printed color-coded labels were introduced. A separate bin of reversal is recommended.
13 Y	Adverse drug reaction	Tachycardia 150–160/min with severe hypotension	Rapid infusion of Inj.Vancomycin, Red Man Syndrome after 10 min	Phenylephrine boluses and rapid fluid given	Be vigilant after medication is known for such side effects*
4 Y	Treatment failure	Laryngospasm resulted in desaturation of up to 72%	An inefficient dose of Atracurium resulted in laryngospasm	Manual ventilation was done with 100% oxygen, and laryngospasm relieved	Brand change for Atracurium requested to the Department of Pharmacy

D = Days; Y = Years; M = Months, STD = supra therapeutic dose, SbTD = Sub therapeutic Dose

*incidents discussed in CI Departmental meeting for awareness of the entire department

**Incidents sent as lessons to learn e-mail to the entire department

Human error was involved in 180 (91.8%) incidents followed by system errors 12 (6%) and equipment errors 4 (2%). Human errors were further classified into lack of knowledge, judgment, or failure to check in 87(48%) reports, nonadherence to standard practice in 52 (29%), stress factor in 22 (12.2%), and lack of proper communication in 19 (10.5%) reports. Furthermore, failure to check was observed in 35/87 (40%), lack of judgment in 34/87 (39%), and lack of knowledge in 18/87 (21%) reports.

In total 2249 reports, the level of harm was broadly categorized into no harm in 169 and harm in 27 reports. Out of 27 (1.2%) errors that caused harm to patients, the severity was observed as significant (*n* = 11), serious (*n* = 15), and life-threatening (*n* = 1). Though it is not easy to cover detailed reports of each ADE but is shown in Tables 2 and 3.

**Table 3:** The cause, immediate and late corrective measures taken in significant ADEs

Age	Type of ADEs	Incident	Cause	Immediate effect/ action	Corrective measures
5 D	STD	Delayed recovery, double dose of Atracurium given	Dose calculation not done	Patients kept ventilated	Adherence to the anesthesia plan and dose calculation*
2 M Pre-term 40 W	STD	Fentanyl syringe kept attached to IV side port and mistakenly used as flush after giving reversal drugs Delayed emergence and extubation.	Deviation from standards	The patient kept ventilated and extubated after an hour	Never keep syringes attached to side ports Color-coded stickers were provided
20 D	STD	Bradycardia from 120 to 74/min As an anesthetist was unable to get proper sleep due to personal reason	Ten times higher dose of Inj. Neostigmine was given, 1 mg instead of 0.1 mg	Inj. Atropine was given and HR increased to 130/per min	In unusual situations, inform the supervisor to ensure patient safety*
5 Y	STD	Patient with low GCS received a triple dose of Narcotic and induction agent.	No thought was given to pathological changes.	---	Calculation of drug doses with clinical correlation*
6 Y	Non-adherence	Forgot to give analgesia at induction resulting in tachycardia after the incision.	Fentanyl missed at I/V induction	I/V analgesic given instantly	Adherence to the anesthesia plan and assigning roles if two operators are present
1 Y	Non-adherence	Unable to induce patient with inhalation induction with Sevoflurane 8%	The selector knob of the ventilator was not turned ON	The selector knob switched to ventilator mode	Quick machine check before every case
11 Y	Non-adherence	Tachycardia and hypertension, the cause was unknown so narcotic and muscle relaxant repeated.	Forgot to turn ON the volatile agent.	A volatile agent started and 5 mls of Propofol given	A quick scan from the machine end to the patient end*
17 Y	Wrong drug	Atracurium was given instead of narcotic, instantly noticed, and sedated. The patient received a higher dose of atracurium but recovered.	Syringe swap, as narcotics and muscle relaxant both were in 5 ml syringes	Thio-pentone sodium was given immediately	Department Policy: Narcotics switched to 10 cc syringes and muscle relaxant in 5 cc syringe
3 Y	Wrong drug	Delayed recovery at the end of oesophageal dilation	Atracurium was given instead of Fentanyl due to a Syringe swap	Patient kept ventilated	Dept policy: Narcotics switched to 10 cc syringes and muscle relaxant in 5 cc syringe
9 Y	Treatment failure	The patient started coughing on laryngoscopy	Inefficacy of Atracurium	Extra dose given and confirmed muscle paralysis before laryngoscopy	Strategies for cold chain maintenance reviewed and reinforced. Refrigerators were placed in the induction room for temperature-sensitive medications.
4 Y	SbTD	A smaller dose of Atropine administered	Lack of knowledge	The desired effect was not achieved and the dose repeated.	Calculation of doses with clinical correlation*

D = Days; Y = Years; M = Months, STD = supra therapeutic dose, SbTD = Sub therapeutic Dose

*incidents discussed for the awareness of the entire department

**Incidents sent as lessons to learn e-mail to the entire department

## Discussion

This review identified and analyzed 196 medication-related incidents (31.5% of the total 622 pediatric critical incidents) between 2001 and 2020 in pediatric surgical patients admitted to our hospital. Paralytic agents, narcotic analgesics, sedatives, and antibiotics added to 70% of the errors. Human error made the highest contribution i.e. 91% to these MEs and 48% of these errors occurred due to lack of mandatory checks, lack of judgment, and knowledge gaps followed by 29% of the errors due to deviation from standard practice. During medication handling, administration contributed to 45% of the reports. The errors that produced harm to the patients were 1.2% and out of these 15 were serious, 11 significant, and one life-threatening. All ADEs were timely managed and did not result in serious morbidity/mortality. Common administrative errors observed by us were repetition (35%), incorrect doses (33%), and substitution (23%). In comparison, Gariel et al. found a much higher incidence of incorrect doses (67.5%), in a prospective study [12]. Woo et al. have also reported an eight-fold higher accidental overdose in children compared to the adult population [13].

The major drug categories involved were neuro-muscular blockers, opioids, and sedatives/hypnotics. This is comparable to the Australian Incident Monitoring Study (AIMS) which was done in adults [14]. Neuromuscular blockers and opioid overdose and under-dose both are detrimental in children. Dilution, concentration, and volume of infusions are also of great concern, especially in neonates. Time and cost constraints, complex environments, fatigue, stress, non-routine events, and many other factors can affect anesthetist performance [15, 17]. We found a high incidence of human error (92%) and the commonest cause was failure to check and failure to judge (34%). Marcus showed that error of judgment was as high as 43% in 668 pediatric anesthetic incidents in humans [16]. The most likely cause of these human errors is that different personnel prepare/dilute and administer medications. When more than one operator is involved in a case there is a higher chance of an error. A system of double checks of preparation, standardized doses, drug dilution, and syringe standardization policy should be in place for the pediatric population [17]. Stress (12.2%), poor communication (10.5%), and lack of knowledge (8.7%) were other causes. Communication errors for medication can be reduced by “closed loop communication”, standardization of oral instructions, and making it a rule to repeat the given instructions [18].

The value of CI reporting is proven in academic, research, planning and development of policies, guidelines, budget, and medication processes as well as the provision of safe anesthesia care [19, 20]. It is a low-cost tool and is of value in healthcare setups of LMICs because other QI measures require a significant monetary investment. CI reporting program was introduced in our department in 1996 and is still one of the regular activities in our departmental quality program. This simple strategy has supported us in improving the standard of anesthetic care in low-resource settings [21].

An adverse drug event is “an injury resulting from the use of a drug. Under this definition, the term ADE includes harm caused by the drug (adverse drug reactions and overdoses) and harm from the use of the drug (including dose reductions and discontinuations of drug therapy)” [22]. Adverse Drug Events may result from medication errors but not every medication error results in adverse events. ADEs include allergy, adverse drug reaction, sub/supra therapeutic dose, treatment failure, drug misuse, drug interaction, drug withdrawal, and non-adherence. Tables 2 and 3 show errors according to the level of harm as well as what happened, what was done immediately, and action plans. The outcomes of such errors are variable and may range from clinically insignificant to a life-threatening event.

Lessons learnt from some selected incidents were discussed, and recommendations were then reinforced as routine practice. Firstly, labels were to be placed lengthwise on the syringe to ease the reading of all information, including concentration, date, time, and Mnemonics of the person preparing the drug, without covering the syringe scale. Secondly, medication ampoules should be broken, drawn, and labelled one at a time to avoid any error. Thirdly, a written medication plan for every pediatric patient should be preoperatively prepared and available on the workstation.

Common reasons for medication preparation errors were incorrect labeling, wrong dilutions, and lack of standardized syringe use. Several steps for improvement were taken between 2007 and 2008. Two such examples were the introduction of pre-printed color-coded labels and syringe standardization. Comparison of MEs before and after this intervention period has shown considerable change in the outcome, resulting in a substantial decline in the frequency of ADEs. The frequency of ADE was 20 and 7, before and after the intervention, respectively.

There are multiple limitations to our study such as voluntary and anonymous reporting, single center, and that it was a retrospective review of data. It was only based on reported incidents, and this increases the risk of missing facts and figures as well as important incidents. The common limitations of CIR like, physician bias, underreporting, lack of denominator, and delayed action after group discussion.

Given the above ADEs, our future directions are to conduct medication safety courses/ workshops for pediatric drug handling, adopt standardized anesthesia workstation and drug trolley, adapt of written medication plan and prescription, and use of drug dilution guide, label and prepare one medicine at a time and proper hand over of medication bin to the reliever. However frequent short audits are needed to observe compliance with these measures and reinforcement of practice in the meetings.

## Conclusions

The medication errors reported in this study have shown a limited number of ADEs and very few were serious and life-threatening. Reinforcement of standard practice in departmental critical incident meetings provides the basis for quality improvement measures. Continuous efforts through discussion in the meetings, patient safety workshops and lessons to learn e-mails were some low-cost strategies to enhance medication safety during anaesthesia.

## Data Availability

The datasets used and/or analyzed during the current study are available from the corresponding author on reasonable request.

## Abbreviations

MEs: Medication Errors
LMIC: Lower Middle-Income Country
ADE: Adverse Drug Event
CIRS: Critical Incidents Reporting System
CI: Critical Incidents
